# Population, Physiological, and Genetic Insights into Carbendazim Resistance in Populations of the Phytopathogenic Fungus *Microdochium nivale*

**DOI:** 10.3390/jof11090639

**Published:** 2025-08-29

**Authors:** Guzalia Sh. Murzagulova, Olga A. Gogoleva, Egor A. Ryazanov, Karina A. Shatravina, Natalia V. Tendiuk, Ildar T. Sakhabutdinov, Sergey N. Ponomarev, Inna B. Chastukhina, Olga N. Makshakova, Mira L. Ponomareva, Vladimir Y. Gorshkov

**Affiliations:** 1 Federal Research Center “Kazan Scientific Center of the Russian Academy of Sciences”, 420111 Kazan, Russia; murzagulova196@gmail.com (G.S.M.); gogolewaoa@yandex.ru (O.A.G.); eg.ryazanov@gmail.com (E.A.R.); shatravina-karina@mail.ru (K.A.S.); natalya.tendyuk@mail.ru (N.V.T.); teilszx@yandex.ru (I.T.S.); s.ponomarev2020@yandex.ru (S.N.P.); innachast@yandex.ru (I.B.C.); omega12@inbox.ru (O.N.M.); smponomarev@yandex.ru (M.L.P.); 2 Institute of Fundamental Medicine and Biology, Kazan Federal University, 420008 Kazan, Russia

**Keywords:** fungicide resistance, intrapopulation heterogeneity of phytopathogenic fungi, *Microdochium nivale*, winter cereal crops, virulence, plant infectious diseases

## Abstract

Fungicide treatment is one of the most common methods for controlling fungal plant diseases. However, many phytopathogenic fungi develop resistance to fungicides. Addressing this agriculturally important issue requires comprehensive investigations into fungicide resistance. Our study aims to assess the degree and prevalence of resistance to carbendazim—one of the most widely used fungicides—in populations of *Microdochium nivale*, the causal agent of the deleterious plant disease pink snow mold; to explore possible relationships between carbendazim resistance and physiological and genetic traits; and to gain insight into the molecular basis of carbendazim resistance in this species. We showed that carbendazim resistance is widespread in the analyzed *M. nivale* populations, and that the application of carbendazim increases the proportion of resistant strains. Nevertheless, carbendazim-resistant strains are present at high relative abundance in populations that have never been exposed to fungicides. Carbendazim resistance in *M. nivale* is strongly associated with sequence variations in the β-tubulin gene, resulting in amino acid sequence variability that leads to differential affinity for carbendazim. Additionally, we propose a metabarcoding-based approach employing a genetic marker linked to a specific phenotypic trait to assess the ratio of genotypes with contrasting properties within a particular fungal species in environmental communities.

## 1. Introduction

Phytopathogenic fungi cause significant losses in agricultural crop yields worldwide. One of the most common methods for controlling them in the field is the application of fungicides. However, the effectiveness of fungicides is continually reduced due to fungal adaptation and the development of fungicide resistance [1,2,3]. The management of fungicide application is an important issue in agriculture, and addressing it requires comprehensive investigations into fungicide resistance in phytopathogenic fungi.

The application of fungicides leads to the emergence within fungal populations of clones and subsequently subpopulations with varying levels of fungicide resistance and diverse genetic backgrounds associated with that resistance. The following major mechanisms of fungicide resistance formation are highlighted: (1) point mutations in the gene encoding the fungicide target that lead to decreased fungicide affinity for its target; (2) overexpression or duplication of the target gene, resulting in increased levels of the target protein; (3) increased production of transporters responsible for fungicide efflux from the cell or prevention of fungicide entry into the cell; (4) metabolism (detoxification) of the fungicide within the fungal cell [1,4,5].

The changes that confer fungicide resistance may affect other fungal phenotypes, such as virulence, growth rate, sporulation, and survival under stress conditions, all of which can influence the fitness of fungi in agrocenoses [6,7,8,9,10,11,12]. Fungicide-resistant subpopulations often have a selective advantage over fungicide-sensitive ones following fungicide application. However, in the absence of fungicide selective pressure, sensitive subpopulations tend to outcompete resistant ones [13]. Herewith, fungicide resistance conferred by different genetic backgrounds may differentially affect fungal fitness in fungicide-free environments [11,13,14,15].

One of the widely used fungicides in agriculture is benzimidazole fungicides [1,16,17,18]. These fungicides inhibit microtubule assembly by binding to β-tubulin [19,20,21]. Due to the widespread application of benzimidazole fungicides, resistance to these fungicides is widely represented in populations of phytopathogenic fungi [16,17,22,23,24]. For example, in populations of *F. graminearum* and *F. asiaticum*, 3% and 8% of strains, respectively, were resistant to carbendazim—a compound from the benzimidazole class [17,25], whereas in populations of *Sclerotinia sclerotiorum*, 69% of strains were carbendazim-resistant [24]. The emergence of resistance to benzimidazole fungicides is often associated with point mutations in the β-tubulin gene [26,27], and alterations in phenotypic traits resulting from the acquisition of carbendazim resistance have been described [7,28].

Fungicides are also recommended for use against one of the most difficult-to-manage plant diseases—snow mold [29,30]. This disease is caused by fungi that exhibit both phytopathogenic and psychrotolerant or psychrophilic properties, parasitizing overwintering plants beneath the snow cover and shortly after its melting [29,31,32,33]. The most widespread causal agent of snow mold is *Microdochium nivale*, which causes pink snow mold as well as other plant diseases throughout the growing season. Since fungicide application is not possible once snow cover is present, fungicides to control snow mold can be used only preventively (before the snow cover forms). The list of fungicides recommended for controlling snow mold is limited. Among them, benzimidazole fungicides, including those based on carbendazim, are the most widely used. The limited list of fungicides and their use prior to disease progression increases the risk of resistance development in *M. nivale* populations.

The resistance of *M. nivale* to imidazole and benzimidazole fungicides, strobilurins, succinate dehydrogenase inhibitors, phenylpyrrole fungicides, and azole fungicides has been described [34,35,36,37,38,39,40,41,42]. However, in most studies, screening of *M. nivale* strains for fungicide resistance was performed at concentrations (~10^−5^–10^−9^ M) significantly lower than those used in agricultural practice (~10^−2^–10^−3^ M). Therefore, although these studies demonstrate intraspecific heterogeneity among *M. nivale* strains regarding their differential sensitivity to various fungicides, it remains debatable whether the described phenotypes can be attributed to truly resistant strains. The mechanism of fungicide resistance in *M. nivale* has been studied only with respect to inhibitors of succinate dehydrogenase, which is associated with mutations in the *sdhB* gene [43].

The aim of the present study was to assess the degree and prevalence of carbendazim resistance in *M. nivale* populations from two agrocenoses in Russia, explore possible relationships between carbendazim resistance and physiological and genetic traits, and gain insight into the molecular basis of carbendazim resistance in this species.

## 2. Materials and Methods

### 2.1. Strains Used in This Study

The 132 *M. nivale* strains used in this study were characterized in terms of their virulence, growth rates, and genetic traits in our recent paper [44]. Briefly, the strains were isolated from samples of three winter cereal crops: winter rye (*Secale cereale* L. cv. Ogonek), winter wheat (*Triticum aestivum* L. cv. Nadezhda), and winter triticale (×*Triticosecale* Wittm. cv. Kornet), all grown under uniform agronomic management (including fertilization and herbicide application) in competitive variety field trials—standardized agricultural experiments designed to compare the performance of multiple crop varieties under identical conditions. Plant samples for isolating *M. nivale* strains were collected one week after snowmelt in 2020 from two agrocenoses located 59 km apart in a straight line within the Republic of Tatarstan: (1) Bolshiye Kaban in the Laishevo district (latitude 55.625164 N, longitude 49.351334 E) and (2) Nalasa in the Arsk district (latitude 56.113468 N, longitude 49.774500 E). The two agrocenoses, hereafter referred to as Arsk and Laishevo, differed in their soil-climatic and agrotechnological conditions (Table S1). In the Arsk agrocenosis, fungicides have never been used. In the Laishevo agrocenosis, fungicides were not applied during the target vegetative period (autumn 2019–summer 2020); however, in the preceding years (2016–2018), crop seeds grown in Laishevo were treated with the benzimidazole fungicide Ferazim (AgroExpertGroup, Moscow, Russia) according to the manufacturer’s instructions prior to sowing.

### 2.2. Evaluation of Fungicide Resistance of the Strains

To screen 132 *M. nivale* strains for carbendazim resistance, the carbendazim-formulated fungicide Ferazim (AgroExpertGroup, Moscow, Russia) was used. Ferazim was added to potato-sucrose agar (1.5% agar) supplemented with 200 µg/mL gentamicin (PSA) to achieve a final carbendazim concentration of 10^−3^ M (191.2 µg mL^−1^). *M. nivale* strains were grown on PSA both with and without (control) the fungicide. This concentration of carbendazim was applied since it is recommended by the manufacturers for plant treatment. To assess the effect of the fungicide, 7 mm diameter mycelial plugs, cut from the periphery of 10-day-old cultures of the studied strains, were placed at the center of Petri dishes containing PSA and PSA with Ferazim. Fungal cultures were grown in darkness at 20 °C for 2 weeks. Each day, two perpendicular radii of each fungal colony were measured, and their average was used to estimate the fungal growth rate. The growth rate was extrapolated from the slope value of the linear part of the growth curve. Each strain was analyzed in three biological replicates under each condition (with and without the fungicide). The effect of the fungicide was expressed as the percentage of growth inhibition observed in its presence.

### 2.3. Assessment of Physiological and Genetic Traits of the Strains

Virulence, growth rates, and sequences of fragments of the internal transcribed spacer 2 (ITS2), elongation factor 1α (EF-1α), and β-tubulin (β-Tub) genes of the 132 *M. nivale* strains were analyzed in our previous study [44]. Here, these data were used to evaluate possible relationships between the carbendazim resistance (analyzed in this study) of the strains and their physiological and genetic traits (analyzed in our previous study [44]). Briefly, the virulence of each strain was assessed on winter cereal crops (winter rye cv. Ogonek, winter wheat cv. Nadezhda, and winter triticale cv. Kornet) using two methods: the whole-plant assay (WPA) and the detached leaf assay (DLA). The WPA was performed on plants grown axenically on ¼ diluted Murashige and Skoog medium without organic carbon. For sterilization, the seeds were first soaked in water for 2 h and then treated with a 5% AgNO_3_ solution for 15 min. After that, the AgNO_3_ solution was removed, and the seeds were washed five times with sterile 1% NaCl solution. Finally, the seeds were rinsed with sterile water. Seeds were germinated for 36 h at 28 °C in darkness. Seedlings were transferred to individual sterile 50 mL glass tubes containing 10 mL of ^1^/_4_ diluted Murashige and Skoog medium without organic carbon. Twelve hours later, the seedlings were infected by placing an 8 mm mycelial plug (cut from the periphery of 7–10 day old cultures grown on PSA) in contact with the seedling. For the control plants, 8 mm plugs of sterile PSA were used instead of the mycelial plugs. Plants were grown at 20 °C with a 16 h light/8 h dark cycle photoperiod for 20 days. At least 20 biological replicates were analyzed for each strain on each crop. The virulence of the strains was analyzed 20 days after plant inoculation and expressed as the reduced root dry weight (RRDW, %) of infected plants compared to control plants, since this parameter allows for more precise differentiation of strains based on their effect on the host plant than visual symptom assessment [45]; a greater reduction in root dry weight indicates higher strain virulence.

For the DLA tests, plants were grown in soil in a glasshouse for 10 days at 20, under a photoperiod of 16 h of light and 8 h of darkness, with a relative humidity of 60%. Three-cm leaf fragments were cut, placed in plastic containers, and infected with the analyzed strains by placing an 8 mm mycelial plug (cut from the periphery of 7–10 day-old cultures grown on PSA) in the center of each leaf segment. Sterile PSA plugs were placed on the control leaf segments. The containers were covered with glass plates and incubated in a Binder chamber 720 MK (E5) (BINDER GmbH, Tuttlingen, Germany) at 20 °C with a 16 h light/8 h dark photoperiod and 60% relative humidity. Disease lesions, measured in mm, were assessed individually in 30 biological replicates at 2-day intervals (4, 6, 8, and 10 days post-inoculation). The virulence of each strain was evaluated by calculating the area under the disease progress curve (AUDPC) according to the formula:

AUDPC = ^1^/_2_ (x_1_ + x_2_) (t_2_ − t_1_) +…+ (x_n−1_ + x_n_) (t_n_ − t_n−1_)

where AUDPC is the area under disease progress curve; n is the number of observations; x_1_, x_2_, x_n_ are the percentage of visible infected area at the time of the first, second, and further registration, %; t_2_ − t_1_ are the time between the second and first registration, days; t_n_ − t_n−1_ are the time between last and next-to-last registration, days.

Nucleotide sequences of the *M. nivale* strains were determined by amplicon sequencing using the Illumina MiSeq platform. The internal transcribed spacer 2 (ITS2), a fragment of the elongation factor 1α gene (EF-1α), and a fragment of the β-tubulin gene (β-Tub) were amplified using primers ITS3_KYO2: 5′–GAT GAA GAA CGY AGY RAA–3′ and ITS4: 5′–TCC TCC GCT TAT TGA TAT GC–3′ [46]); MicEF_F1: 5′–GGT GAG TTC GAG GCT GGT ATC–3′ and MicEF_R1: 5′–CAG GGG GCG TTG GTG G–3′ [44]; MicTub_F1: 5′–ACG CTC CTC ATC TCC AAG ATC–3′, MicTub_R1: 5′–GAA ACG CAG GCA GGT GGT–3′ [44], respectively. All DNA libraries were prepared according to the Illumina protocol (Illumina protocol, part no. 15044223, Rev. B). Amplification of the target fragments was carried out using Q5 high-fidelity DNA polymerase (NEB, Ipswich, MA, USA). The PCR products were purified with AMPure XP magnetic beads (Beckman Coulter, Brea, CA, USA). The indexing of libraries was performed using the Nextera XT Index Kit v2 (Illumina, San Diego, CA, USA), followed by purification of the indexed libraries with AMPure XP magnetic beads. The libraries were sequenced using the MiSeq Reagent Kit v3 (600-cycles) (Illumina). The sequences were deposited in the NCBI database (accession numbers PQ516916.1, PQ516917.1, PQ538502.1, PQ538503.1, PQ538504, PQ538505, PQ538506).

### 2.4. Metabarcoding

For metabarcoding, specifically to determine the ratios between different *M. nivale* β-Tub sequences in samples of winter cereal crop roots and dead parts of shoots (DPS), we used DNA samples from our recent study that characterized the total fungal and bacterial communities (based on ITS2 and 16S rRNA amplicon sequencing) of winter cereal crops [47]. The same plant samples were also used for isolating the *M. nivale* strains analyzed in this study. To determine the ratios between different *M. nivale* β-Tub sequences, samples of wheat roots were excluded because the *M. nivale* content in these samples was negligible (based on the relative abundance of *M. nivale* ITS2 sequences) [47]. Additionally, some replicates of other sample types with negligible *M. nivale* content were also excluded. As a result, this study analyzed the following number of replicates in each category with respect to the relative abundance of *M. nivale* β-Tub sequences: Arsk—wheat DPS (7), rye DPS (8), triticale DPS (8), rye root (8), and triticale root (6); Laishevo—wheat DPS (7), rye DPS (8), triticale DPS (8), rye root (8), and triticale root (8).

Primers for the amplification of *M. nivale* β-Tub (MicTub_F1: 5′–ACG CTC CTC ATC TCC AAG ATC–3′, MicTub_R1: 5′–GAA ACG CAG GCA GGT GGT–3′) were designed in our previous study to ensure amplification of a fragment of less than 450 bp that includes single-nucleotide polymorphisms reflecting the variability of the target loci at the intraspecific level [44]. DNA libraries were prepared following the Illumina protocol (Illumina protocol, part no. 15044223, Rev. B). The target *M. nivale* β-Tub fragment was amplified using Q5 High-Fidelity DNA Polymerase (NEB, Ipswich, MA, USA). The PCR products were purified with AMPure XP magnetic beads (Beckman Coulter, Indianapolis, IN, USA). The indexing of libraries was performed using the Nextera XT Index Kit v2 (Illumina, San Diego, CA, USA), followed by purification of the indexed libraries with AMPure XP magnetic beads. The libraries were then pooled and sequenced on the MiSeq platform using the MiSeq Reagent Kit v3 (600-cycles) (Illumina). All datasets were deposited in the National Center for Biotechnology Information (NCBI) Sequence Reading Archive (SRA) and are available under the PRJNA1285676 bioproject.

The obtained reads were processed for quality control and primer sequence removal using FastQC [48], MultiQC [49], and Cutadapt v.3.5 [50]. The DADA2 pipeline [51] was subsequently used for quality trimming, dereplication, chimera filtering, and the generation of amplicon sequence variants (ASVs). Taxonomic assignment of the generated ASVs was performed using NCBI BLAST. The three most abundant ASVs corresponding to the *M. nivale* β-tubulin gene region were retained for analysis. The relative abundance of these three ASV variants (designated A, B, and C) was then calculated for each sample, with their combined raw read counts representing 100%.

### 2.5. Molecular Docking

The spatial structure of the *M. nivale* β-tubulin protein was built using homology modeling in Modeller 9.15 [52,53]. Chicken β-tubulin class II (PDB: 5CA1), sharing 97% similarity with *M. nivale* β-tubulin, was used as a template. The coordinates of carbendazim were derived from those of its analogue, nocodazole (PDB: 5CA1). Molecular docking of carbendazim to three variants of *M. nivale* β-tubulin protein, each differing by a single amino acid substitution, was performed using AutoDock4 [54,55]. The grid was restricted to the binding pocket where the identified amino acid substitutions were located. The generation of initial positions was performed using a Genetic Algorithm with a population size of 150 and 27,000 generations, resulting in a total of 2,500,000 energy evaluations. The binding energy was calculated as the sum of contributions from van der Waals contacts, hydrogen bonds, desolvation energy, electrostatic energy, and torsional entropy. Final docking poses were clustered using a 2.0 Å cutoff.

### 2.6. Statistics

Pearson’s χ^2^ test (*p*-value < 0.05) was used to evaluate whether two agrocenoses significantly differ in terms of the ratios between carbendazim-resistant and carbendazim-sensitive strains, or the ratios between strains with different variants of barcodes (ITS2, EF-1α, β-Tub), as well as to assess the association between carbendazim resistance and the origin of the strains (crop or plant part from which the strains were isolated). Fisher’s exact test (*p*-value < 0.05) was used to evaluate whether two agrocenoses, different crops, or different plant parts differ in the ratios between strains with carbendazim resistance-related and carbendazim sensitivity-related β-Tub variants. The significant difference in growth rate or virulence between carbendazim-resistant and carbendazim-sensitive strains was assessed using the Mann–Whitney test (*p*-value < 0.05).

## 3. Results

### 3.1. Resistance and Sensitivity of Microdochium nivale Strains to Carbendazim

**Figure 1:**
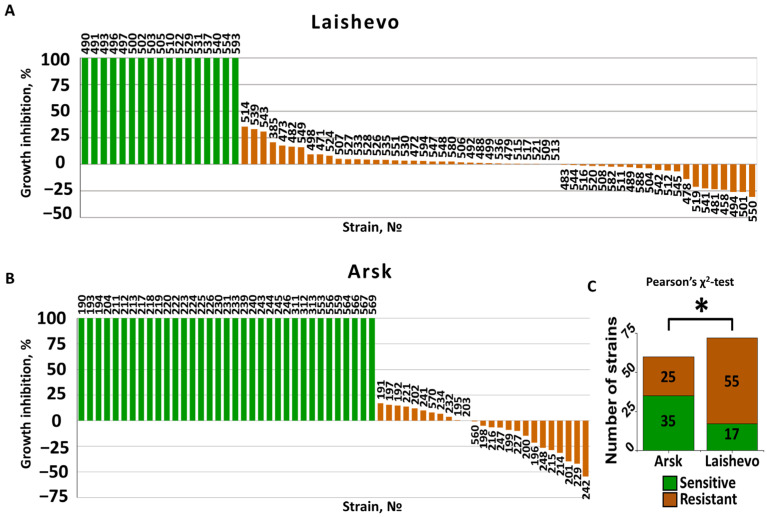
Sensitivity (green columns) and resistance (brown columns) of 132 *Microdochium nivale* strains to carbendazim. Panels (**A**,**B**) show the distribution of growth inhibition by carbendazim for strains isolated from the Laishevo and Arsk agrocenoses, respectively. A 100% growth inhibition indicates sensitivity of the strain to carbendazim, whereas negative values indicate that carbendazim stimulated the growth of the strain. Numbers above or below the columns show the last three digits of the strain’s accession number. Panel (**C**) shows the results of a χ^2^ test, indicating that the proportion of resistant strains within the strain pool was statistically higher (asterisk, *p* < 0.05) in the sample of strains from the Laishevo agrocenosis compared to that in the sample of strains from the Arsk agrocenosis.

Out of the 132 analyzed *M. nivale* strains, 80 (61%) displayed resistance to carbendazim (10^−3^ M or 191.2 µg mL^−1^). The growth of sensitive strains was completely inhibited (100%) by carbendazim (Figure 1A,B). Resistant strains either showed similar growth rates in the presence and absence of the fungicide, or displayed less than 40% growth inhibition, or even exhibited growth stimulation by carbendazim, resulting in negative growth inhibition values (Figure 1A,B). The growth of sensitive, but not resistant, strains was also completely inhibited at a carbendazim concentration 10 times lower (10^−4^ M). In the sample of strains isolated from the Laishevo agrocenosis, which had previously been treated with benzimidazole fungicides, the proportion of resistant strains was statistically higher than in the sample of strains isolated from the Arsk agrocenosis, where fungicides had not been previously applied (Figure 1C). Specifically, 76% (55 out of 72) of strains from Laishevo were resistant, whereas 42% (25 out of 60) of strains from Arsk showed resistance to carbendazim.

### 3.2. Analysis of Potential Relationships Between Carbendazim Resistance and Various Phenotypic Traits of M. nivale Strains

No relationships between the carbendazim resistance and virulence were observed, regardless of the host plant (rye, wheat, triticale) or the method (WPA and DLA) used for virulence assays (Table S2). The growth rates of carbendazim-resistant and carbendazim-sensitive *M. nivale* strains did not differ either (Table S3). Resistant strains were isolated significantly more frequently from rye, whereas sensitive strains were significantly more prevalent among those isolated from triticale (Pearson’s χ^2^-test, *p* < 0.05) (Table S4). No relationship was found between carbendazim resistance and the plant part from which the strains were isolated (Pearson’s χ^2^-test, *p* < 0.05) (Table S4).

### 3.3. Analysis of Potential Relationships Between Carbendazim Resistance and Genetic Traits of M. nivale Strains

The analyzed *M. nivale* strains were previously divided into different phylogenetic groups based on the nucleotide sequences of three barcodes: internal transcribed spacer 2 (ITS2), elongation factor 1α (EF-1α), and β-tubulin (β-Tub) [44]. Within 132 analyzed strains, 83 and 49 strains had “A” and “B” ITS2 variants, respectively (PQ516916.1 and PQ516917.1); 80 and 52 strains had “A” and “B” EF-1α variants, respectively (PQ538502.1 and PQ538503.1); and 52, 75, and 5 strains had “A”, “B”, and “C” β-Tub variants, respectively (PQ538504, PQ538505, and PQ538506). In the present study, we examined the potential association between the attribution to phylogenetic groups (barcode variants) and resistance to carbendazim.

**Figure 2:**
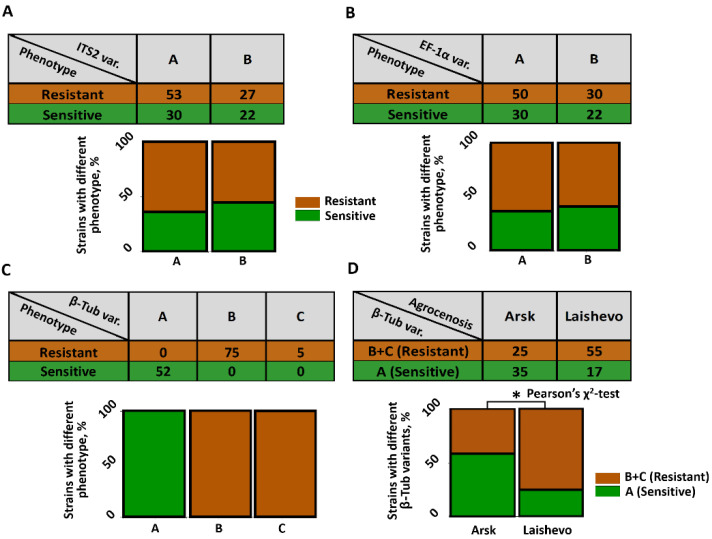
Distribution of 132 *Microdochium nivale* strains into distinct genotypes and phenotypes for the analysis of the association between carbendazim resistance and nucleotide sequences of three barcodes: internal transcribed spacer 2 (ITS2) (**A**), elongation factor 1α (EF-1α) (**B**), and β-tubulin (β-Tub) (**C**). The association between resistance and sequence variants was assessed using Pearson’s χ^2^-test (*p* < 0.05). The numbers in the contingency tables show the actual number of strains in each category. The graphs display the percentage distribution of resistance (brown)/sensitivity (green) within each barcode variant. Panel (**D**) shows the results of a χ^2^ test, indicating that the proportion of strains with the “B” and “C” β-Tub variants together (associated with carbendazim resistance) within the strain pool was statistically higher (asterisk, *p* < 0.05) in the sample of strains from the Laishevo agrocenosis compared to that in the sample of strains from the Arsk agrocenosis.

The frequency of carbendazim resistance among strains with the “A” ITS2 variant was similar to that for the strains with the “B” ITS2 variant (Pearson’s χ^2^-test, *p* < 0.05) (Figure 2A). Similarly, carbendazim-resistant and carbendazim-sensitive strains were comparably represented within the groups of strains carrying the “A” and “B” variants of EF-1α (Pearson’s χ^2^-test, *p* < 0.05) (Figure 2B). In turn, a strong relationship was observed between carbendazim resistance and the β-Tub sequence variant. All 52 strains with the “A” β-Tub variant were carbendazim-sensitive, while all strains with the “B” (75 strains) and “C” (5 strains) β-Tub variants were carbendazim-resistant (Figure 2C). Herewith, in the sample of strains isolated from the Laishevo agrocenosis, which had been previously treated with benzimidazole fungicides, the proportion of strains with the “B” and “C” β-Tub variants together (associated with carbendazim resistance) within the strain pool was statistically higher than that in the sample of strains isolated from the Arsk agrocenosis, where fungicides had not been previously applied (Figure 2D).

### 3.4. Relative Abundance of Different M. nivale β-Tubulin Gene Sequences in Environmental Plant Samples

**Figure 3:**
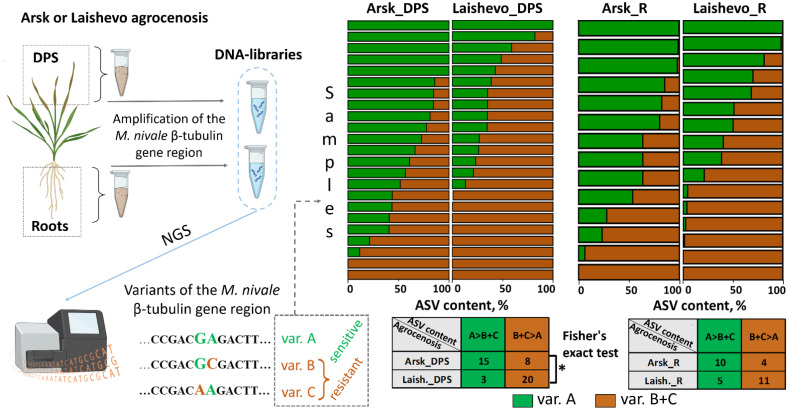
The comparison of the relative abundance of different *Microdochium nivale* β-tubulin gene sequence variants (corresponding to carbendazim sensitivity or carbendazim resistance of the strain) in environmental plant samples (roots (R) or dead parts of shoots (DPS)) collected from two agrocenoses: one previously treated with benzimidazole fungicides (Laishevo) and one untreated (Arsk). The charts show the relative abundance of amplicon sequence variants (ASVs) corresponding to β-tubulin gene variants associated with carbendazim sensitivity (variant A, green) and carbendazim resistance (variants B and C together, brown) in each analyzed sample. The numbers in the contingency tables show the number of samples with a predominance of either carbendazim sensitivity-related ASVs (green) or carbendazim resistance-related ASVs (brown). The significance of the difference (asterisk, *p* < 0.05) in the relative abundance of carbendazim sensitivity-related ASVs and carbendazim resistance-related ASVs in different agrocenoses was analyzed using Fisher’s exact test. NGS—next-generation sequencing.

The ratios between carbendazim sensitivity-related (A variant) and carbendazim resistance-related (B and C variants together) β-Tub sequences were calculated and compared between two agrocenoses and three crops, separately for root and DPS samples (Figure 3, Table S5).

Within the untreated Arsk agrocenosis, only 8 of 23 DPS samples had a higher proportion of carbendazim resistance-related sequences than carbendazim sensitivity-related sequences. In contrast, within the fungicide-treated Laishevo agrocenosis, 20 of 23 DPS samples exhibited a higher proportion of carbendazim resistance-related sequences compared to carbendazim sensitivity-related sequences (Figure 3). Fisher’s exact test confirmed that the proportion of carbendazim resistance-related sequences was significantly higher in Laishevo DPS samples compared to Arsk DPS samples.

A similar pattern was observed in the root samples. Within the Arsk agrocenosis, 4 of 14 root samples had a higher proportion of carbendazim resistance-related sequences than carbendazim sensitivity-related sequences. Within the Laishevo agrocenosis, 11 of 16 root samples showed a higher proportion of carbendazim resistance-related sequences than carbendazim sensitivity-related sequences. Although there was a trend toward a higher proportion of carbendazim resistance-related sequences in Laishevo root samples compared to Arsk root samples, this difference was not statistically significant according to Fisher’s exact test. Resistance-related *M. nivale* β-Tub sequences were observed significantly more frequently in rye DPS samples (81%), whereas sensitivity-related sequences were significantly more common in triticale DPS samples (63%) (Fisher’s exact test, *p* < 0.05) (Table S5).

### 3.5. Effect of Identified Single-Nucleotide Polymorphisms (SNPs) in the M. nivale β-Tubulin Gene on the Affinity of β-Tubulin Protein for Carbendazim

**Figure 4:**
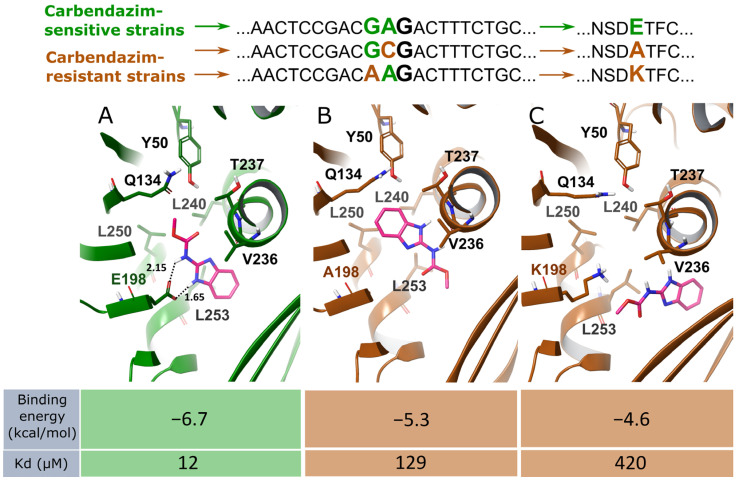
Modeling of carbendazim interactions with different variants of *Microdochium nivale* β-tubulin differing in the amino acid residues at position 198. Variant A (E198) of β-tubulin is typical of carbendazim-sensitive strains, whereas variants B (A198) and C (K198) are typical of carbendazim-resistant strains. E198 facilitates the formation of hydrogen bonds with carbendazim (**A**), which are absent in variants A198 (**B**) and K198 (**C**). K198 leads to electrostatic repulsion between carbendazim and the carbendazim-binding pocket of β-tubulin (**C**).

The β-Tub sequences in the studied *M. nivale* strains contain single-nucleotide polymorphisms (SNPs) at codon 198 (accession numbers PQ538504, PQ538505, and PQ538506; Figure 3 and Figure 4). These SNPs result in variations in the amino acid sequence of the target protein: glutamic acid residue (E198) in variant A, which is associated with carbendazim sensitivity; and alanine (A198) and lysine (K198) residues in variants B and C, respectively, both associated with carbendazim resistance. We proposed that the identified amino acid substitutions might alter the affinity of the *M. nivale* β-tubulin protein for carbendazim, thereby resulting in either resistance or sensitivity to the fungicide in different *M. nivale* strains. To verify this hypothesis, molecular docking of carbendazim to different variants of *M. nivale* β-tubulin was performed. The structure of the carbendazim–β-tubulin complex has not been experimentally resolved. Therefore, in our study, the coordinates of carbendazim within *M. nivale* β-tubulin were derived from those of its structural analogue, nocodazole, within the nocodazole–chicken β-tubulin complex, which is the most closely related experimentally resolved complex to the target one (PDB: 5CA1) [56].

Docking of carbendazim to β-tubulin (E198) yielded two clusters, with the ligand oriented toward the bottom of a narrow binding pocket via either the 1,3-benzimidazole ring or the methyl ester group. In the former case, the cluster was poorly populated (~20%); therefore, this orientation of carbendazim within the protein’s binding pocket was considered a false positive. In turn, the latter cluster, reflecting ligand orientation toward the binding pocket via the methyl ester group, was highly populated (~80%) and the complex was characterized by a low binding energy (−6.7 kcal/mol), corresponding to a dissociation constant (Kd) of approximately 12 μM (Figure 4). In addition, this orientation of carbendazim within β-tubulin (E198) was similar to the experimentally resolved orientation of nocodazole in the binding site of chicken β-tubulin (PDB: 5CA1) [56]. Therefore, this orientation of carbendazim within the protein’s binding pocket was deemed correct. Within the carbendazim–β-tubulin (E198) complex, (1) residues L240, L250, L253, V236, T237, Q134, E198 and Y50 were located at distance of less than 4 Å from the ligand and formed the surface of the ligand-binding pocket; (2) the NH groups of carbendazim formed hydrogen bonds with the carboxyl moiety of E198 in β-tubulin at O···H distances of 1.65 and 2.15 Å; (3) and the leucine residue (L253) supported hydrophobic interactions with the aromatic moiety of carbendazim (distance between L253 and the ligand: 3.06 Å) (Figure 4).

The substitution of the glutamic acid residue (E198) with an alanine residue (A198) in *M. nivale* β-tubulin abolished hydrogen bonding between the NH groups of carbendazim and β-tubulin and yielded five clusters, three of which were moderately populated (20–40%) and characterized by higher binding energies (minimum −5.3 kcal/mol) compared to those observed in the carbendazim–β-tubulin (E198) complex (−6.7 kcal/mol) (Figure 4). The substitution of the negatively charged glutamic acid residue (E198) with the positively charged lysine residue (K198) in β-tubulin led to electrostatic repulsion between the ligand and the protein, resulting in a rather superficial ligand position within the protein’s binding site; this type of complex had a binding energy of −4.6 kcal/mol (Figure 4).

Thus, our results demonstrate that β-tubulin variants with alanine or lysine residues at position 198, typical of carbendazim-resistant *M. nivale* strains, exhibit lower affinity for carbendazim (with a dissociation constant an order of magnitude higher) compared to β-tubulin variants with a glutamic acid residue at position 198, typical of carbendazim-sensitive *M. nivale* strains. Therefore, these substitutions provide a clear explanation of the mechanisms underlying carbendazim resistance in *M. nivale*.

## 4. Discussion

In the present study, we examined the degree and prevalence of carbendazim resistance in two Russian *M. nivale* populations, explored the relationship between carbendazim resistance and the physiological and genetic traits of the strains, and investigated the molecular basis of carbendazim resistance in this species. Carbendazim resistance appeared to be widespread in the analyzed *M. nivale* populations: more than half of the tested strains were resistant to this fungicide. Such a high prevalence of carbendazim resistance has also been previously observed in *Sclerotinia sclerotiorum* parasitizing oilseed rape [24], *Fusarium graminearum* isolated from cereal crops [57], *Corynespora cassiicola* originating from cucumber [58,59], and *Colletotrichum fructicola* causing peach anthracnose [60].

Among *M. nivale* strains isolated from the agrocenosis previously treated with benzimidazole fungicides (Laishevo), carbendazim-resistant strains were more prevalent than among strains isolated from the untreated agrocenosis (Arsk). This indicates that, first, the use of fungicides increases the proportion of resistant strains relative to sensitive ones. A similar trend in carbendazim resistance has also been previously reported for phytopathogens such as *Fusarium graminearum* [17], *Zymoseptoria tritici* [61], *Oculimacula yallundae* [62], *O. acuformis*, and *Ustilaginoidea virens* [7]. As a result, the development of resistance to benzimidazole fungicides is considered to depend on the frequency of their application [16,17,22,23,24,63,64]. Second, even in the agrocenosis where fungicide treatment had not been applied previously, carbendazim-resistant *M. nivale* strains were present. This phenomenon has also been observed in *Colletotrichum gloeosporioides* and *Botrytis cinerea* [65,66,67]. The presence of carbendazim-resistant strains in high proportions within untreated agrocenoses (as well as high proportions of carbendazim resistance-related *M. nivale* β-Tub sequences in environmental plant samples) likely implies the following: (1) carbendazim-resistant strains can spread across wide territories; (2) the absence of the selective factor (the fungicide) does not necessarily lead to a significantly lower survival advantage for carbendazim-resistant *M. nivale* strains in agrocenoses compared to carbendazim-sensitive *M. nivale* strains. Therefore, the carbendazim-resistant phenotype can be maintained in global populations. The latter statement is consistent with the absence of differences in virulence and growth rates between the samples of carbendazim-resistant and carbendazim-sensitive *M. nivale* strains in our study. The absence of an effect of carbendazim resistance acquisition on virulence and growth rate has also been observed in *Sclerotinia sclerotiorum* and *B. cinerea* [11,13,14]. However, in some cases, strains resistant to carbendazim or other benzimidazole fungicides differ from fungicide-sensitive strains in certain physiological traits, such as increased production of the mycotoxin deoxynivalenol (DON) in *Fusarium graminearum* [28] and reduced growth rate and conidiogenesis in *Ustilaginoidea virens* [7]. At the same time, we found that carbendazim-resistant *M. nivale* strains were isolated significantly more frequently from rye, whereas carbendazim-sensitive strains were significantly more commonly isolated from triticale. To our knowledge, variations in the proportions of fungicide-resistant versus fungicide-sensitive strains of phytopathogens with a broad host range, depending on the host plant species, have not been previously demonstrated.

In our previous paper, the studied *M. nivale* strains were divided into different phylogenetic groups based on the sequences of ITS2, EF-1α, and β-Tub [44]. In the present study, we examined whether strains of different phylogenetic groups differ in their predisposition to acquire carbendazim resistance. No association was found between ITS2 or EF-1α variants and the target phenotype. In turn, a strong (100%) relationship was revealed between the β-Tub variant and the target phenotype: all strains with the “A” β-Tub variant were carbendazim-sensitive, while all strains with the “B” and “C” β-Tub variants were carbendazim-resistant. This is evidently related to the fact that β-tubulin, in addition to being a phylogenetic marker, is a target of carbendazim.

As discussed above, a higher proportion of strains with carbendazim resistance (and resistance-related β-Tub variants) were isolated from the carbendazim-treated agrocenosis than from the untreated one, implying that the use of this fungicide promotes the accumulation of fungicide-resistant strains. However, this statement is based on the results of indirect culture-based methods, which cannot reliably reflect the quantitative distribution of strains within the environmental community, since culture conditions may provide a selective advantage to particular phenotypes and thus distort the actual situation occurring in the agrocenosis. To verify the observed pattern, we took advantage of a metabarcoding approach based on next-generation sequencing (NGS), which provides an objective, culture-independent picture of the microbial community. Usually, metabarcoding is used to determine the quantitative composition of the total fungal community based on the distribution of ASV variants of ITS2, a universal barcode for the overwhelming majority of fungi that enables their taxonomic differentiation during analysis [68]. Additionally, since the ITS2 sequence does not enable differentiation of some higher-rank taxa (e.g., genera), some studies have used additional barcodes to further resolve particular taxa into lower-rank taxonomic groups (e.g., species) [69]. In our study, we used the identified genetic marker (β-Tub) of the target phenotypic trait (carbendazim resistance) as a barcode in metabarcoding to directly verify the patterns observed with culture-based approaches, namely that the resistant genotypes are more prevalent in the Laishevo (benzimidazole-treated) agrocenosis and rye plants, whereas the sensitive genotype is more prevalent in the Arsk (benzimidazole-untreated) agrocenosis and triticale plants.

The results of the barcoding were consistent with those obtained using the culture-based method. In DPS samples from plants grown in benzimidazole-treated agrocenosis (Laishevo), the proportion of resistance-related β-Tub sequences was significantly higher than in corresponding samples from fungicide-untreated agrocenosis (Arsk). A similar trend was observed for root samples; however, it was not supported by statistical significance. Lower expression of the differential abundance of resistance- and sensitivity-related β-Tub sequences in roots compared to DPS is possibly due to the fact that the selective pressure of the fungicide is less pronounced inside living plant tissues (roots) than in dead plant remnants. Metabarcoding data also confirmed that rye DPS were more intensively colonized by carbendazim-resistant *M. nivale* genotypes, whereas triticale DPS were predominantly colonized by carbendazim-sensitive genotypes.

To the best of our knowledge, this study is the first to use a genetic marker of a specific phenotypic trait in NGS-based metabarcoding of fungal communities to assess the ratio of genotypes of a particular fungal species with contrasting properties within environmental communities. A similar approach has been used recently, but with respect to nematodes inhabiting animal feces [70]. In nematodes (*Haemonchus* spp.), the β-tubulin gene also contains SNPs associated with benzimidazole resistance. Using this genetic marker, as well as a marker for resistance to another anthelmintic, levamisole, the authors showed that benzimidazole resistance is more prevalent than levamisole resistance in nematodes [70]. Additionally, the β-tubulin gene sequence has previously been used in metabarcoding of fungal communities; however, this barcode was employed for phylogenetic purposes, specifically to differentiate species within the *Colletotrichum* genus (which is not possible through analysis of ITS2 sequences) [71], rather than as a marker of a phenotypic trait to assess the ratio of genotypes of a particular species with contrasting properties, as in our study.

Thus, the metabarcoding-based approach proposed in our study enables obtaining an objective assessment of the prevalence of carbendazim resistance in *M. nivale* populations. This approach is more precise and less time-consuming than microbiological methods for assessing carbendazim resistance within natural *M. nivale* populations. The elucidation of genetic markers of resistance to a range of fungicides in the most devastating phytopathogens will provide the foundation for developing a ‘panel’ of barcodes to be used in agricultural practice, yielding valuable information to support fungicide management planning.

The revealed association between variants of the β-Tub sequence and carbendazim resistance in *M. nivale* has also been previously widely described for other fungal species [19,20,21]. This is expected, as β-tubulin is the target of carbendazim [26,27]. In different phytopathogens, carbendazim resistance can be associated with SNPs at various positions and different nucleotide substitutions within a particular codon, leading to varying levels of resistance [16,72,73]. The SNPs in the *M. nivale* β-Tub gene identified in our study, which are associated with differential resistance to carbendazim, result in polymorphism at amino acid position 198: glutamic acid (E198) residue in variant A, associated with carbendazim sensitivity, and alanine (A198) and lysine (K198) residues in variants B and C, respectively, both associated with carbendazim resistance. SNPs at codon 198, among SNPs associated with carbendazim resistance, are considered the most widespread in phytopathogens and are thought to have no effect on physiological traits, including virulence [10,12,17,19,23,25,58,63,66,74,75]. Herewith, SNPs resulting in alternative amino acid residues at position 198 have been associated with different levels of carbendazim resistance in *B. cinerea* and *Fusarium* species [25,72,74]. Additionally, different amino acid residues conferring carbendazim resistance appear to reduce fungal fitness in the environment to varying degrees. In *B. cinerea*, resistant strains with valine at position 198 exhibited reduced fitness compared to resistant strains with alanine or lysine at the same position, and in the absence of fungicide, all three carbendazim-resistant variants were replaced by the carbendazim-sensitive variant, which exhibited the highest fitness [13]. In our study, carbendazim-resistant *M. nivale* strains carrying the A198 β-tubulin variant (B variant of the β-Tub sequence) were unlikely to have a significant survival disadvantage in agrocenoses compared to carbendazim-sensitive *M. nivale* strains with the E198 β-tubulin variant (A variant of the β-Tub sequence), since both strain types (and both β-Tub sequence types) were found in roughly comparable proportions within the agrocenosis that had not been treated with fungicides. At the same time, carbendazim-resistant *M. nivale* strains with the K198 β-tubulin variant (C variant of the β-Tub sequence) were rarely isolated, accounting for only 5 out of 132 strains, with four of those being collected from the agrocenosis where carbendazim had been applied. Whether the “rarity” of this genotype is associated with its reduced competitiveness compared to the other two genotypes requires further investigation.

Although the nucleotide sequence at codon 198 has been shown to be associated with carbendazim resistance in a wide range of phytopathogenic fungi, the effect of amino acid variation at the corresponding position on carbendazim resistance has been analyzed in a more limited number of species (*Fusarium* species, *Monilinia fructicola*, *Botrytis cinerea*, *Podosphaera xanthii*, *Alternaria solani*, *Corynespora cassiicola*) [10,17,23,63,66,74,75,76,77]. Since the structure of the carbendazim–β-tubulin complex has not been experimentally resolved, the interactions between fungal β-tubulins and carbendazim have been modeled in silico using either blind docking or targeted docking. When blind docking was employed, the carbendazim-binding sites often did not coincide with the sites of the amino acid polymorphism; therefore, it has been suggested that the amino acid polymorphisms affected the 3D structures of β-tubulins, including the carbendazim-binding sites, leading to reduced affinity for carbendazim [76,77,78].

Targeted docking has previously been performed based on the structure of the nocodazole–chicken β-tubulin complex, which is the most closely related experimentally resolved complex to the carbendazim–fungal β-tubulin complex [79]. Amino acid substitutions associated with carbendazim resistance are known to be located in the nocodazole-binding site of β-tubulin, and mutations at this site in yeast confer resistance to both nocodazole and carbendazim [80,81,82]. These facts indicate that nocodazole and carbendazim share similar binding sites in β-tubulin; therefore, when targeted docking is used, the carbendazim-binding site coincides with the site of the amino acid polymorphism. We considered that targeted docking is more reliable than blind docking, and by using it, we have shown that *M. nivale* β-tubulin variants with alanine or lysine residues at position 198, typical of carbendazim-resistant *M. nivale* strains, exhibit lower affinity for carbendazim compared to β-tubulin variants with a glutamic acid residue at position 198, typical of carbendazim-sensitive *M. nivale* strains. Thus, these substitutions provide a clear explanation of the mechanisms underlying carbendazim resistance in *M. nivale*. A similar tendency has been previously described for phytopathogenic fungi such as *Botrytis cinerea* and *Monilinia fructicola* [75,83].

## 5. Conclusions

Carbendazim resistance is widespread in the studied *M. nivale* populations from Russia. The application of benzimidazole fungicides increases the proportion of carbendazim-resistant strains relative to sensitive strains in *M. nivale* populations. *M. nivale* strains are present at high relative abundance in agrocenoses where fungicide treatment has not previously been applied, indicating that (1) carbendazim-resistant strains can spread across wide territories, and (2) the absence of the selective factor (the fungicide) does not necessarily lead to a significantly lower survival advantage for carbendazim-resistant *M. nivale* strains in agrocenoses compared to carbendazim-sensitive *M. nivale* strains. Carbendazim resistance in *M. nivale* is strongly associated with the sequence characteristics of the β-tubulin gene. An NGS-metabarcoding-based approach using a genetic marker linked to a specific phenotypic trait is proposed to assess the ratio of genotypes with contrasting properties in a particular fungal species within environmental communities. β-Tubulin variants carrying alanine or lysine residues at position 198, characteristic of carbendazim-resistant *M. nivale* strains, exhibit lower affinity for carbendazim than variants with a glutamic acid residue at this position, typical of carbendazim-sensitive strains, thereby providing a clear explanation of the mechanisms underlying carbendazim resistance in *M. nivale*.

## Data Availability

Sequencing data from this study can be found in the National Center for Biotechnology Information (NCBI) Sequence Reading Archive (SRA) under the PRJNA1285676 bioproject.

## Supplementary Materials

The following supporting information can be downloaded at: https://www.mdpi.com/article/10.3390/jof11090639/s1, Table S1: Comparison of the two agrocenoses considered in the present study; Table S2: The virulence of carbendazim-resistant and carbendazim-sensitive *Microdochium nivale* strains toward three winter crops (rye, wheat, or triticale); Table S3: The growth rates of carbendazim-resistant and carbendazim-sensitive strains of *Microdochium nivale*; Table S4: Evaluation of the relationships between carbendazim resistance of *Microdochium nivale* strains and their origin using the Pearson’s χ^2^-test; Table S5: Evaluation of the relationships between the relative abundance of different *Microdochium nivale* β-tubulin gene sequence variants (corresponding to carbendazim sensitivity (A variant) or carbendazim resistance (B and C variants together) of the *M. nivale* strains) and the type of analyzed samples using the Fisher’s exact test.
